# Oral administration of *Faecalibacterium prausnitzii* and *Akkermansia muciniphila* strains from humans improves atopic dermatitis symptoms in DNCB induced NC/Nga mice

**DOI:** 10.1038/s41598-022-11048-4

**Published:** 2022-05-05

**Authors:** Yoonmi Lee, Hye Rim Byeon, Seo-Yul Jang, Moon-Gi Hong, Dohak Kim, Dokyung Lee, Joo-Hyun Shin, Yesol Kim, Seung-Goo Kang, Jae-Gu Seo

**Affiliations:** 1 R&D Center, Enterobiome Inc., 814 Siksa-dong, Ilsandong-gu, Goyang-si, 10326 Korea; 2 Institute of Bioscience and Biotechnology, Kangwon National University, Chuncheon, Korea

**Keywords:** Microbiology, Skin diseases

## Abstract

Atopic dermatitis (AD) is a common inflammatory skin disease, and its pathogenesis is closely associated with microbial homeostasis in the gut, namely the gut-skin axis. Particularly, recent metagenomics studies revealed that the abundance of two major bacterial species in the gut, *Faecalibacterium prausnitzii* and *Akkermansia muciniphila*, may play a critical role in the pathogenesis of AD, but the effect of these species in AD has not yet been elucidated. To evaluate the potential beneficial effect of *F. prausnitzii* or *A. muciniphila* in AD, we conducted an animal model study where *F. prausnitzii* EB-FPDK11 or *A. muciniphila* EB-AMDK19, isolated from humans, was orally administered to 2,5-dinitrochlorobenzene (DNCB)-induced AD models using NC/Nga mice at a daily dose of 10^8^ CFUs/mouse for six weeks. As a result, the administration of each strain of *F. prausnitzii* and *A. muciniphila* improved AD-related markers, such as dermatitis score, scratching behavior, and serum immunoglobulin E level. Also, the *F. prausnitzii* and *A. muciniphila* treatments decreased the level of thymic stromal lymphopoietin (TSLP), triggering the production of T helper (Th) 2 cytokines, and improved the imbalance between the Th1 and Th2 immune responses induced by DNCB. Meanwhile, the oral administration of the bacteria enhanced the production of filaggrin in the skin and ZO-1 in the gut barrier, leading to the recovery of functions. Taken together, our findings suggest that *F. prausnitzii* EB-FPDK11 and *A. muciniphila* EB-AMDK19 have a therapeutic potential in AD, which should be verified in humans.

## Introduction

Atopic dermatitis (AD) is a chronic inflammatory disease that commonly occurs in infants and children, often persisting into adulthood^1,2^. The prevalence of AD is reportedly higher in developed countries, indicating a close link to environmental factors, such as pollution and lifestyle changes^3,4^. Being still unknown, the pathogenesis includes complicated interactions contributed by genetic, immunological, and environmental factors^5^. Recently, many studies have shown that dysbiosis of the gut microbiome may be associated with an increased risk of AD^6^.

In the pathogenesis of AD, allergen exposure results in the release of inflammatory cytokines by T helper 2 (Th2) cells, inducing an imbalance between Th1 and Th2 cells, thereby increasing the production of immunoglobulin E (IgE)^7^. These responses are initially driven by thymic stromal lymphopoietin (TSLP), which subsequently triggers a dendritic cell-mediated Th2 inflammatory response^8^. Particularly, acute AD lesions have a marked increase in the number of cells expressing Th2 cytokines, such as IL-4, IL-5, and IL-13, whereas the expression of a significant Th1 cytokine, IFN-γ, is decreased^9^. Clinical studies have shown that serum levels of IL-4, IL-6, and IL-18 were significantly higher in AD patients and associated with the clinical severity of AD^10,11^. Although topical steroids are currently used as the main treatment for AD, they have serious side effects such as dry skin, reduced skin thickness, increased blood pressure, and decreased kidney function due to long-term use. Therefore, it is necessary to develop a safe and effective treatment for AD, from infants to the elderly. The human-derived microbiome research for AD should develop as the safer and more effective AD treatment.

In humans, the gut microbiome plays an essential role in metabolic and immune response^12^. It protects the intestine against colonization by exogenous pathogens and potentially harmful native microbes through several mechanisms, including direct competition for limited nutrients and the modulation of host immune responses^12^. The metabolite from intestinal dysbiosis, which is a condition of microbial imbalance, can access the circulatory system and accumulate in the skin, impairing epidermal differentiation and skin barrier integrity, ultimately negatively affecting skin function^13^. Moreover, intestinal dysbiosis increases epithelial permeability, leading to activation of effector T cells, disrupting the balance with immunosuppressive regulatory T cell^14^. In addition, Kim et al. observed statistical significance of a higher incidence of skin disease in patients with inflammatory bowel disease (IBD), and that suggested that intestinal dysbiosis was associated with an increased risk of inflammatory skin disease^15^. Recent studies have shown a correlation between intestinal dysbiosis and AD^16^. In a clinical study, the metagenomic analysis of fecal samples from AD patients showed a significant reduction of *Faecalibacterium prausnitzii* species compared to control patients and a decrease in the fecal short chain fatty acids (SCFAs), especially butyrate, were observed among the AD patients^17^.

*F. prausnitzii* is an obligate anaerobe residing in the human gut where it reportedly constitute about 5% of the gut microbiota in healthy adults^18^. The species is a butyrate producer and affects physiological functions and homeostasis to maintain health^18^. Its anti-inflammatory activity is well established, and the alteration in the abundance of *F. prausnitzii* in the gut are reportedly associated with several human diseases: a lower abundance in patients with IBD, such as ulcerative colitis (UC) and Crohn’s disease (CD), and colorectal cancer (CRC), compared to healthy subjects^19,20^. Meanwhile, *Akkermansia muciniphila* is also an obligate anaerobe and a mucin-degrading bacterium extensively present in healthy human intestinal mucosa, constituting up to 3% of the gut microbiota^21,22^. It produces SCFAs, mainly propionate and acetate, and is able to modulate the host’s metabolism, immune responses, and gut barrier functions^23,24^. Meanwhile, recent studies have reported that the abundance of *F. prausnitzii* and *A. muciniphila* were reduced in the gut microbiota of children with allergic asthma and AD, respectively^25,26^. The secreted metabolites of these bacteria may induce anti-inflammatory and prevent pro-inflammatory cytokines^25^. However, the clinical effects of these bacteria have not been fully elucidated.

In this study, we evaluated the effects of *F. prausnitzii* and *A. muciniphila* in NC/Nga mice with DNCB-induced AD-like symptoms. Our findings indicate that the oral administration of strains of *F. prausnitzii* or *A. muciniphila* can ameliorate AD-like symptoms by inducing the balance between the Th1 and Th2 cytokines and modulating skin homeostasis and intestinal barrier functions in a DNCB-induced AD model.

## Materials and method

### Ethical approval

The fresh human fecal samples were obtained from each volunteer, and written informed consent was obtained for participants or their parents according to the legal guidance. This research approved by the Korea National Institute for Bioethics Policy (No. P01-201705-31-002), Republic of Korea. It was conducted in accordance with the ‘Research Ethics Regulation’ stipulated by the National Bioethics Policy Institute, Korea.

The animal study was approved by the ‘Institutional Animal Care and Use Committee’ of the Dongguk University, Korea (IACUC-2020-002-1) and conducted in compliance with the “Guide for the Care and Use of Laboratory Animals” (Institute for Laboratory Animal Resources, Commission on life sciences, National Research Council of The National Academies, USA; The National Academies Press: Washington, DC, 1996). The germ-free mice study was approved by the ‘Institutional Animal Care and Use Committee’ of the POSTECH University, Korea (POSTECH-2021-0111) and conducted in accordance with the guidelines of the Institutional Animal Care and Use Committee of POSTECH. All experimental were performed in accordance with the ARRIVE guidelines (http://arriveguidelines.org).

### *F. prausnitzii* and *A. muciniphila* bacterial strains

Fecal samples were collected from healthy Koreans aged between 7 and 60 years with approval from the ‘Institutional Review Board of Dongguk University Ilsan Hospital (2018-06-001-012). Although the abundance of *A. muciniphila* and *F. prausnitzii* can be changed according to age, some reports described that they were found in a wide range of ages^22,27^. Therefore, there was no age restriction for collecting the fecal samples to isolate the species with genotypic and phenotypic diversity. The *F. prausnitzii* strains were isolated from the fecal samples according to the previous method with some modifications^28^. Briefly, the EOS (extremely oxygen sensitive) colonies from each sample were obtained on a culture medium (herein referred to as YBHI), brain–heart infusion medium supplemented with 5 g/L yeast extract, 1 g/L cellobiose, 1 g/L maltose, and 0.5 g/L l-cysteine. A species-specific PCR for *F. prausnitzii* (forward primer: 5′-ACTCAACAAGGAAGTGA-3′, reverse primer: 5′-AATTCCGCCTACCTCTG-3′) was done to identify strains of the species, producing a PCR product of 192 bp. Finally, 16S rRNA gene sequencing was performed after PCR amplification using primers 27 F (5′-AGAGTTTGATCCTGGCTCAG-3′) and 1492 R (5′-GGTTACCTTGTTACGACTT-3′). The viable isolates were stocked at − 80 °C with 20% glycerol. The reference strain of *F. prausnitzii* used as a control in this study was obtained from the DSMZ-German Collection of Microorganism and Cell Cultures (DSM17677, strain designation A2-165). All *F. prausnitzii* strains were grown at 37 °C in YBHI medium in an anaerobic chamber filled with 90% N_2_, 5% CO_2,_ and 5% H_2_. *A. muciniphila* type strain BAA-835 (ATCC BAA-835) and *A. muciniphila* EB-AMDK19 (KCTC13761BP) have been previously described^29,30^. They were cultured in soy-peptone-based medium containing (L^−1^): 20 g soy-peptone; 10-g yeast extract; 2.5-g K_2_HPO_4_; 5-g *N*-acetyl-d-glucosamine; 5-g d-lactose; 2.5-g d-fructose; 2.5-g sodium caseinate; 8-g l-aspartic acid; 0.1-mg cyanocobalamin; 0.5-g l-cysteine hydrochloride in an anaerobic chamber filled with 90% N_2_, 5% CO_2,_ and 5% H_2_ at 37 °C. After cultivation, the bacterial cells were pelleted at 12,000*g* for 5 min at 4 ℃, washed with sterile phosphate-buffered saline (PBS) followed by centrifugation. Then they were resuspended in PBS, aliquoted, and stored at − 80 ℃ until needed, respectively. *F. prausnitzii* or *A. muciniphila* suspended in 150-μL anaerobic PBS (1.0 × 10^8^ colony-forming units per mouse) was orally administered to mice.

### Genome analysis and genome-to-genome identity

The whole genomes of *F. prausnitzii* EB-FPDK11 and *A. muciniphila* EB-AMDK19 were sequenced using the PacBio RS II (Pacific Biosciences, Menlo Park, CA, USA) sequencing platform. The sequenced reads were assembled using HGAP 3.0^31^ with a 3 Mb expected genome size. Chromosome circularization and correction of the genome-start position were performed using the tool Circlator^32^. Determination and annotation of the functional genes were conducted using the NCBI Prokaryotic Genome Annotation Pipeline (PGAAP)^33^. The genomic contents of *F. prausnitzii* and *A. muciniphila* were downloaded from the NCBI genome database (http://www.ncbi.nlm.nih.gov/genome/) and the genomic distance was estimated by a whole genome comparison method known as average nucleotide identity (ANI). The ANI values between genomes were computed using pyani with default options^33^.

### Animals

Six-week-old male NC/Nga (weighed about 21–25 g), (SLC, Inc., Japan) were purchased from Daehan Biolink Co. Ltd. (Korea). All mice were housed in as 12-h light/dark cycle under a constant temperature of 20 ± 3 °C, humidity of 55 ± 5%. All mice were supplied with a basal diet and sterilized water without any restrictions during the experiment. The study was approved by the ‘Institutional Animal Care and Use Committee’ of the Dongguk University (IACUC-2020-002-1) and conducted in strict accordance with the recommendations of the “Guide for the Care and Use of Laboratory Animals” (Institute for Laboratory Animal Research, Committee for the Update of the Guide for the Care and Use of Laboratory Animals, National Research Council of The National Academies, USA; The National Academies Press: Washington, DC, USA, 2011). After acclimatization, the animals were randomly divided into seven groups as follows: Normal control group (Normal), DNCB (1-chloro-2,4-dinitrobenzene) with PBS (DNCB), DNCB with dexamethasone (DEX), DNCB with *F. prausnitzii* A2-165 (A2-165), DNCB with *F. prausnitzii* EB-FPDK11 (EB-FPDK11), DNCB with *A. muciniphila* BAA-835 (BAA-835), and DNCB with *A. muciniphila* EB-AMDK19 (EB-AMDK19). Each experimental group consisted of nine mice. The A2-165, EB-FPDK11, BAA-835, and EB-AMDK 19 groups were administered the respective bacterial strains orally in sterile PBS at a daily dose of 1 × 10^8^ CFUs per mouse for six weeks. The Normal and DNCB groups were administered sterile PBS as a vehicle instead of bacterial strains. For the DEX group, as a positive control, dexamethasone was diluted with distilled water to 60 μg/mL and administered orally at 200 μL daily.

The germ free 8-week-old female C57BL/6 mice were bred and maintained in sterile flexible film isolators (Class Biological Clean Ltd.) by feeding autoclaved Teklad global 18% protein rodent diets (2018S, Envigo, USA) in the animal facility of POSTECH Biotech Center as previous study^34^.

### Induction of Atopic dermatitis model

After acclimatization for a week, the dorsal hair of all NC/Nga mice was removed using an electronic clipper and hair removal cream before atopic dermatitis-like skin lesion induction by DNCB treatment. The DNCB (1-chloro-2,4-dinitrobenzene, Sigma, St. Louis, USA) solution was prepared at a concentration of 1% in an acetone:olive oil suspension (3:1), and repeated challenge was performed in the dorsal skin of mice twice a week for 3 weeks. During the EB-bacterial strains administration, mice were challenged with 0.5% DNCB twice a week.

### Evaluation of skin lesions

The severity of dermatitis score was evaluated once a week after three weeks of DNCB treatment. The severity of (1) erythema/hemorrhage, (2) scarring/dryness, (3) edema, and (4) excoriation/erosion was scored as 0 (none), 1 (mild), 2 (moderate), and 3 (severe). Dermatitis score was defined as the sum of these individual scores.

### Frequency of scratching

Scratching behavior was observed after all treatments were completed. The mice were placed into cages and were acclimated for 1 h, and then scratching behavior was counted for 10 min. The method used for behavioral observations was a modification of Kim et al^35^. The scratching behavior score was measured three times by three investigators who were blind to the experiment. To distinguish between scratching and grooming, use of hind toes only was counted as scratching.

### Histological analysis

The dorsal skins, ears, and large intestine tissues of the experimental mice were removed on the final day of the study and fixed in 10% phosphate-buffered formalin. Tissues were embedded in paraffin sliced into 4-μm thick sections and stained with hematoxylin and eosin (H&E). For histological grading in the skin, epithelial hypertrophy and hyperkeratosis of each mouse were scored as follows: 0, normal thickness; 1, two times normal thickness; 2, three times normal thickness; 3, four times normal thickness; or 4, greater than four times normal thickness. The skin sections were stained with Toluidine Blue and Congo Red for counting mast cell and eosinophils. The large intestine sections were stained with Alcian blue PAS (periodic acid-Schiff) for counting goblet cell per intestinal crypt. The observation and analysis of sample images were conducted using a Nikon Eclipse Ni. Morphometric analysis was performed using the ImageJ v.1.51 (National Institutes of Health, Bethesda, MD, USA). All histological examinations were analyzed in six sections/animal slices.

### Immunohistochemistry

For immunohistochemistry staining, the skin and large intestine sections were treated with 3% H_2_O_2_ for 10 min to block endogenous peroxidase activity and incubated with blocking buffer containing 2% bovine serum albumin. Then, the sections were incubated with the primary antibodies, followed by incubation with the biotinylated secondary antibodies. The following primary antibodies were used: anti-Filaggrin (1:100 dilution, #ENZ-ABS181, ENZO, USA), anti-TSLP (1:100 dilution, #ab196990, Abcam, USA), anti-ZO-1 (1:200 dilution, #61-7300, Invitrogen, USA), anti-IL-4 (1:100 dilution, #SC-53084, Santa Cruz Biotechnology, USA), and anti-Claudin-1 (1:100 dilution, #SC-166338, Santa Cruz Biotechnology, USA). Lastly, the color was revealed using 3,3′-diaminobenzidine (DAB). For counting staining, tissue sections were stained with H&E. The observation and analysis of sample images were conducted using a Nikon Eclipse Ni (Nikon Corporation, Japan). For the quantitative analysis, images of three samples from each group were analyzed using the Nikon NIS-Elements image software.

### Total serum IgE and cytokines

At the end of the 9-week treatment period, the mice were sacrificed. Blood samples were collected by heart puncture into heparinized tubes, and the serum was collected by centrifugation at 2000*g* for 20 min at 4 °C. IgE, IL-4, IL-6, IL-10, IL-12 or IFN-γ levels were determined using the mouse ELISA kits (IgE; 88-50460-86, IL-4; 88-7044-86, IL-6; 88-7064-86, IL-10; 88-7105-86, IL-12p70; 88-7121-86, IFN-γ; 88-7316-86, Invitrogen, USA).

### Cellular RNA extraction and quantitative polymerase chain reaction (qPCR)

The total RNA was isolated from the intestines using TRIzol reagent (Life Technologies, Carlsbad, CA, USA) according to the manufacturer’s instructions. First-strand cDNA synthesis from the total RNA template was performed using M-MLV cDNA Synthesis Kit (Enzynomics, Daejeon, Korea). The resulting cDNA was subjected to real-time PCR using qPCR 2 × PreMIX SYBR (Enzynomics, Daejeon, Korea) and a QuantStidio3 (Applied Biosystems, TermoFisher Scientific, MA, USA). The expression level of genes was normalized with the housekeeping gene GAPDH. Use the following primers: murine GAPDH, forward, 5′-AGG TCG GTG TGA ACG GAT TTG-3′, reverse, 5′-AGG TTT GAT TCA GGC AGA TGT T-3′; murine IL-6, forward, 5′-TAG TCC TTC CTA CCC CAA TTT CC-3′, reverse, 5′-TTG GTC CTT AGC CAC TCC TTC-3′; murine IL-13, forward, 5′-CCT GGC TCT TGC TTG CCT T-3′, reverse, 5′-GGT CTT GTG TGA TGT TGC TCA-3′; murine IL-10, forward, 5′-ATT TGA ATT CCC TGG GTG AGA AG-3′, reverse, 5′-CAC AGG GGA GAA ATC GAT GAC A-3′; murine ZO-1, forward, 5′-TTT TTG ACA GGG GGA GTG G-3′, reverse, 5′-TGC TGC AGA GGT CAA AGT TCA AG-3′; human IL-8, forward, 5′-TTT TGC CAA GGA GTG CTA AAG A-3′, reverse, R: 5′-AAC CCT CTG CAC CCA GTT TTC-3′.

### Short chain fatty acids (SCFA) analysis

For SCFA analysis, the culture was centrifuged at 12,000×*g* for 5 min and the supernatant was collected. The concentration of acetate, butyrate, propionate in the medium before inoculation and in the supernatant of the culture was measured by gas chromatography (GC). The procedure was repeated, at least, 3 times for each sample. Chromatographic analysis was carried out using an Agilent 7890N GC system equipped with FFAP column (30 m × 0.320 mm, 0.25 µm phase) as previous study^36^. The conditions were shown in Supplementary Table S2. The oven temperature was maintained at 40 °C for 2 min and then ramped to 240 °C. Injection was performed at 240 °C; the injection volume was 2 μL with split ratio 20:1. The detector gases were air and hydrogen; their flow rates were regulated at 350 and 40 mL/min, respectively.

### Statistical analysis

All statistical analyses were performed suing the GraphPad Prism software (GraphPad Software, La Jolla, CA, USA). The results of multiple group analysis were analyzed using one-way analysis of variance followed by Turkey’s significant difference test. The results were expressed as the mean ± standard error of means (SEM). *P* value of less than 0.05 was considered significant. (***P < 0.001, **P < 0.01, *P < 0.05).

## Results

### Bacterial strains of *F. prausnitzii *and *A. muciniphila*

We selected *F. prausnitzii* EB-FPDK11 from our *F. prausnitzii* strain library consisting of ~ 80 different strains, isolated from healthy Koreans, based on, e.g., (1) anti-inflammatory activity using human cell lines such as HT-29^37^, (2) TEER (transepithelial electric resistance) value using Caco-2 cell line, and (3) the immunomodulating activity based on the IL-10 and IL-12 production using BMDCs from mice (Fig. S1)^38,39^. The *A. muciniphila* EB-AMDK19 strain was described in the previous study^29^. The electron microscopy images of both strains are shown in Fig. S2. We performed whole-genome sequencing to characterize the genomic features of the strains. The genomic features of both strains along with the respective type strains (*F. prausnitzii* ATCC 27768 [type strain of phylogroup I], *F. prausnitzii* A2-165 [type strain of phylogroup II], and *A. muciniphila* BAA-835) are shown in supplementary Table S1. Briefly, *F. prausnitzii* EB-FPDK11 had a genome size of approximately 2.8 Mb and 2579 coding sequences (CDSs), which was smaller than those of the type strains (Table S1 and Fig. S3A). The ANI analysis indicated that *F. prausnitzii* EB-FPDK11 had an 84.7% and 97.5% sequence identity with ATCC 27768 and A2-165, respectively (Fig. S4).

Meanwhile, the genome size of *A. muciniphila* EB-AMDK19 was approximately 2.7 Mb and had 2195 CDSs, which were larger than those of the type strain (Table S1 and Fig. S3B). The ANI analysis of *A. muciniphila* EB-AMDK19 showed a 97.6% sequence identity with ATCC BAA-835.

### Effects of the oral administration of *F. prausnitzii* and *A. muciniphila* on alleviating DNCB-induced AD-like symptoms in NC/Nga mice

**Figure 1:**
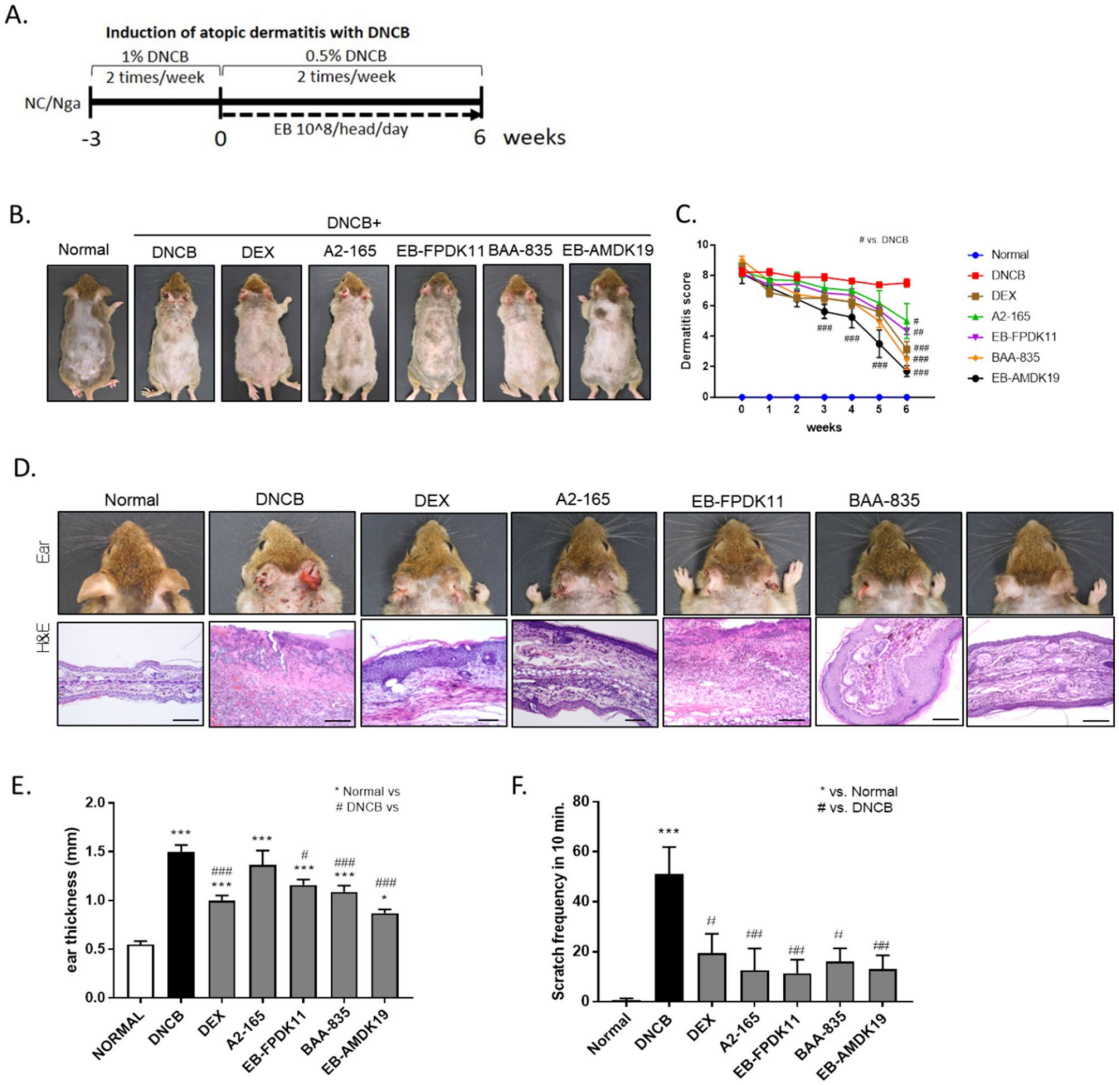
EB-FPDK11 and EB-AMDK19 efficiently prevent the symptoms of AD-like lesion in NC/Nga mice. (**A**) Schematic diagram of the study protocol. (**B**) Photograph of the dorsal skin lesions. (**C**) Dermatitis score were evaluated weekly. (**D**) Photograph and H&E staining of the ear skin lesions from groups, scale bar: 0.1 mm. (**E**) Ear thickness was measured. (**F**) Scratch behavior of the mice was observed for 10 min after sensitization. *P < 0.05; **P < 0.01; ***P < 0.001; *versus normal group; ^#^versus DNCB group.

To investigate if EB-FPDK11 or EB-AMDK19 administration could beneficially affect the AD model, we treated all the experimental groups, except for the normal group, twice weekly with DNCB during the study period on the ear and dorsal skin of the NC/Nga mice (Fig. 1A). After 3 weeks, the treatment groups received dexamethasone (DEX), *F. prausnitzii* A2-165 (A2-165), *F. prausnitzii* EB-FPDK11 (EB-FPDK11), *A. muciniphila* BAA-835 (BAA-835), or *A. muciniphila* EB-AMDK19 (EB-AMDK19) for another 6 weeks, by which time AD-like lesions had developed in the model, including edematous erythema with scratching behaviors, dryness, and excoriation (Fig. 1B–F). The 3-week pretreatment with DNCB caused the development of severe dermatitis, scoring approximately 8 in all the experimental groups (Fig. 1B, C). Hardly any changes in the dermatitis score during the 6-week treatment period were observed in the DNCB group, while the scores of the groups receiving dexamethasone or the respective bacterial strains at a daily dose of 10^8^ CFUs significantly decreased compared with the DNCB group (DEX: 51%, *P* < 0.001; A2-165: 32%, *P* < 0.05; EB-FPDK11: 34%, *P* < 0.01; BAA-835: 53%, *P* < 0.001; EB-AMDK19: 69%, *P* < 0.001).

Histologically, DEX, EB-FPDK11, BAA-835, and EB-AMDK19 administration largely attenuated the pathologic changes in the ear skin lesions, such as hyperkeratosis and inflammatory cell infiltration, compared with the DNCB group, but there were no apparent changes in the A2-165 group (Fig. 1D). Similarly, significant reductions in ear thickness were observed in the treatment groups, except for the A2-165 group (normal: 0.55 ± 0.03 mm, DNCB: 1.50 ± 0.07 mm, EB-FPDK11: 1.16 ± 0.06 mm, *P* < 0.05; BAA-835: 1.1 ± 0.06 mm, *P* < 0.001; EB-AMDK19: 0.87 ± 0.04 mm, *P* < 0.001 compared with the DNCB group) (Fig. 1E). Meanwhile, scratching behavior was counted to assess the effects on itchy skin. The repeated topical application of DNCB resulted in a 7.5-fold-increase in scratching behavior in the normal group (*P* < 0.001) (Fig. 1F). In contrast, scratching behavior was significantly reduced in all the treatment groups, with the EB-FPDK11 and EB-AMDK19 groups showing a 76.2% reduction (*P* < 0.01) and 72.9% reduction (*P* < 0.01), respectively, compared with the DNCB group.

### Effects of *F. prausnitzii* and *A. muciniphila* treatments on IgE level and inflammatory cell infiltration in NC/Nga mice

**Figure 2:**
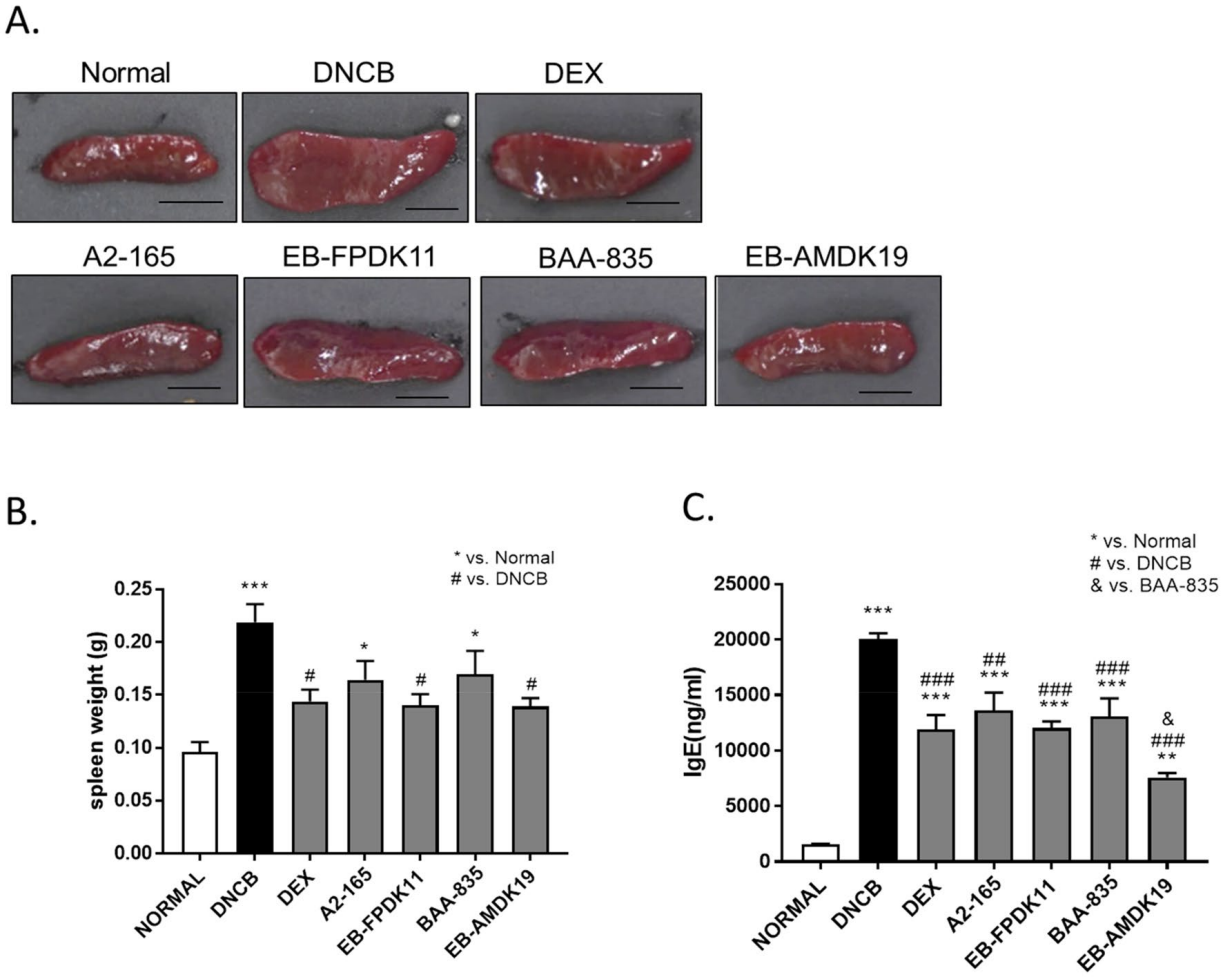
Effect of EB-FPDK11 and EB-AMDK19 on spleen and the total serum IgE levels in DNCB-induced AD-like mice. (**A**) Photograph of spleen from each group of mice to examine morphological alteration. Scale bar, 5 mm. (**B**) The spleen weight of 7 groups at 6 weeks after DNCB treatments. (**C**) Serum level of IgE was measured using ELISA. Data are presented as mean ± SEM of changes in values. *P < 0.05; **P < 0.01; ***P < 0.001, *versus normal group; ^#^versus DNCB group; ^$^versus DEX group; ^&^versus BAA-835 group.

We performed an atopic animal model experiment by applying 1% DNCB twice a week for 3 weeks, and then applying 0.5% DNCB twice a week for 6 weeks. This environment may induce local and systemic immune responses, accordance with Nedoszytko et al.^40^ AD not only causes skin lesion locally, but also causes systemic responses that affect internal tissues and organs such as spleen, bone marrow, and lymph nodes. The spleen contains a range of immune cells and plays an essential role in regulating the immune response^41^. Because splenic enlargement or splenomegaly indicates abnormal immune system function, we investigated whether EB-FPDK11 or EB-AMDK19 administration impacted immune system function. The spleen weight was significantly increased by 2.3-fold in the DNCB group compared with the normal group (*P* < 0.001), which was significantly curbed by treatment with dexamethasone, the EB-FPDK11strain, or EB-AMDK19 strain compared with the DNCB group (*P* < 0.05) (Fig. 2A, B).

One of the most notable characteristics of AD development is an increased level of serum IgE. All the treatment groups showed a significant reduction in serum IgE levels compared with the DNCB group (*P* < 0.001). Notably, the EB-AMDK19 group showed the most substantial reduction in serum IgE, which was even significant when compared with that of the BAA-835 group (EB-AMDK19: 7572 ± 395.3 ng/mL vs. BAA-835: 13,044 ± 1663 ng/mL, *P* < 0.05) (Fig. 2C).

**Figure 3:**
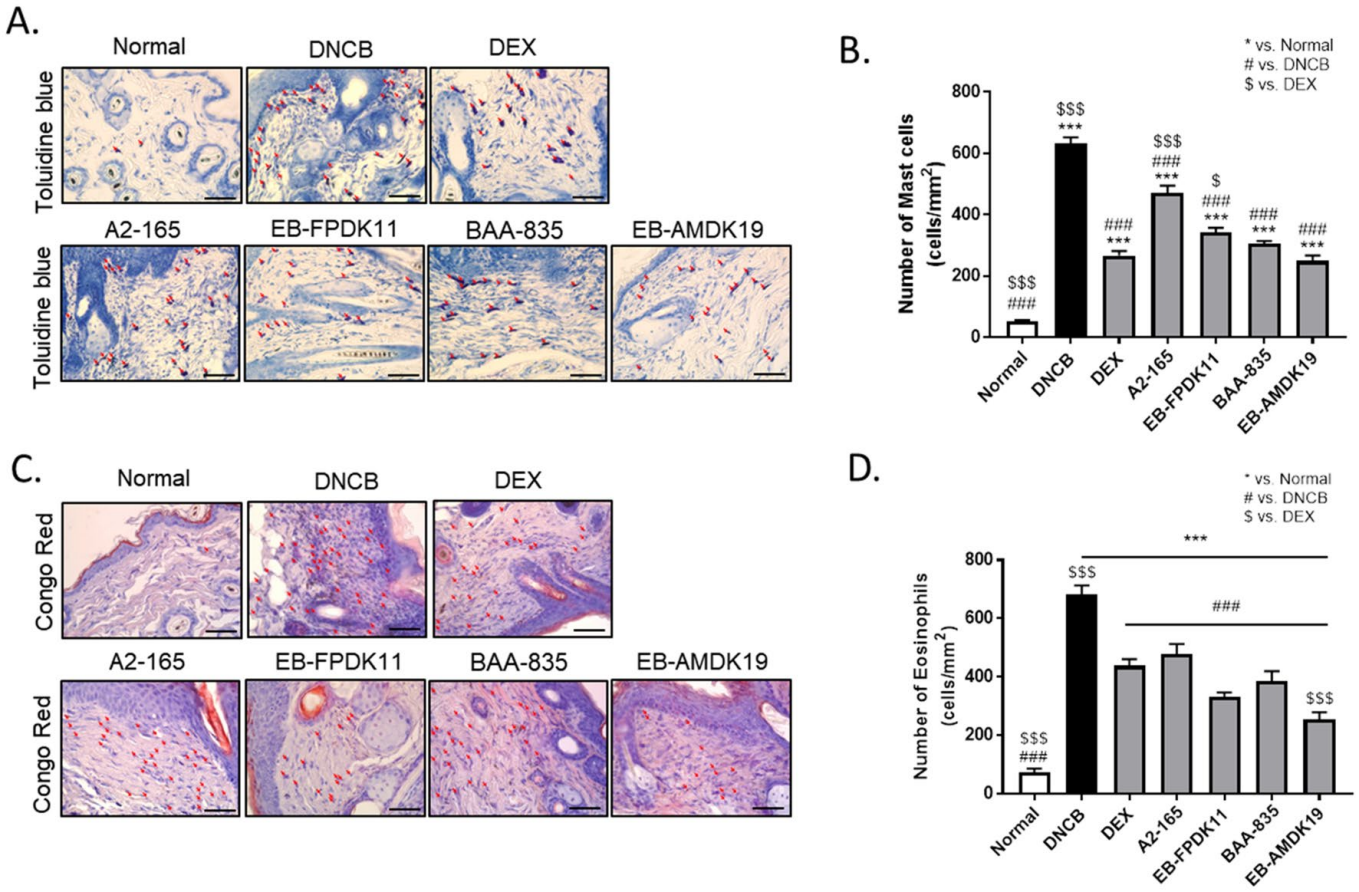
EB-FPDK11 and EB-AMDK19 inhibit mast cell and eosinophil infiltration into dermis in DNCB-induced AD-like mice. (**A**, **B**) Mast cell infiltration in the dorsal skin of mice. Mast cells were stained with Toluidine Blue. Scale bar, 100 µm. (**C**, **D**) Eosinophil infiltration in the dorsal skin mice. Eosinophils were stained with Congo Red. Scale bar, 100 mm. The red arrows indicate positively stained cells. Data are presented as mean ± SEM of changes in values. *P < 0.05; **P < 0.01; ***P < 0.001, *versus normal group; ^#^versus DNCB group; ^$^versus DEX group.

Next, we investigated local infiltration by mast cells. Histamine, released by activated mast cells, allows immune cell infiltration into tissue by increasing blood vessel permeability of blood vessels^42^. Thus, to reduce the allergic response in autoimmune diseases such as AD, it is necessary to control activated mast cells^43^. We treated the dorsal skin tissues of each group with Toluidine Blue and Congo red solutions to stain mast cells and eosinophils, respectively, for examining the effect of DNCB-induced infiltration of mast cells and eosinophils into skin lesions in the treatment groups. The number of inflammatory cells infiltrated in the dermis layer increased following a topical treatment with DNCB, and the fold increases of mast cells and eosinophils were 13.0 ± 1.3 (*P* < 0 0.001) and 14.7 ± 3.9 (*P* < 0.001), respectively, compared with the normal group (Fig. 3A–D). The infiltration of mast cells and eosinophils was significantly reduced in the treatment groups compared with the DNCB group (*P* < 0.001) (Fig. 3). There was no significant difference between the DEX group and the BAA-835 or EB-AMDK19 group in the number of mast cells (Fig. 3A, B). Interestingly, the EB-AMDK19 group showed the largest reduction in the number of eosinophils, which was even significantly lower than the DEX group (*P* < 0.001) (Fig. 3C, D). Therefore, our findings indicated that the administration of *A. muciniphila* and *F. prausnitzii* strains suppressed the DNCB-induced infiltration of eosinophils and mast cells into the skin lesions, among which EB-AMDK19 appeared to be the most efficacious.

### Improvements in damaged skin condition in NC/Nga mice treated with *F. prausnitzii* and *A. muciniphila* strains

AD pathophysiology involves skin barrier and immune function abnormalities. Impaired epidermal tight junction (TJ) proteins, such as ZO-1, claudin-1, and occludin could explain many consequences of skin barrier dysregulation, for example, decreased levels of TJ proteins in AD patients^44^. Filaggrin is a protein derived from pro-filaggrin by keratinocytes. It plays a major structural and functional role in the epidermis, contributing to skin homeostasis^45,46^. It is also crucial for the development of AD and allergic disease^47^, and its expression was significantly reduced in patients with AD, even without filaggrin mutation^48^.

**Figure 4:**
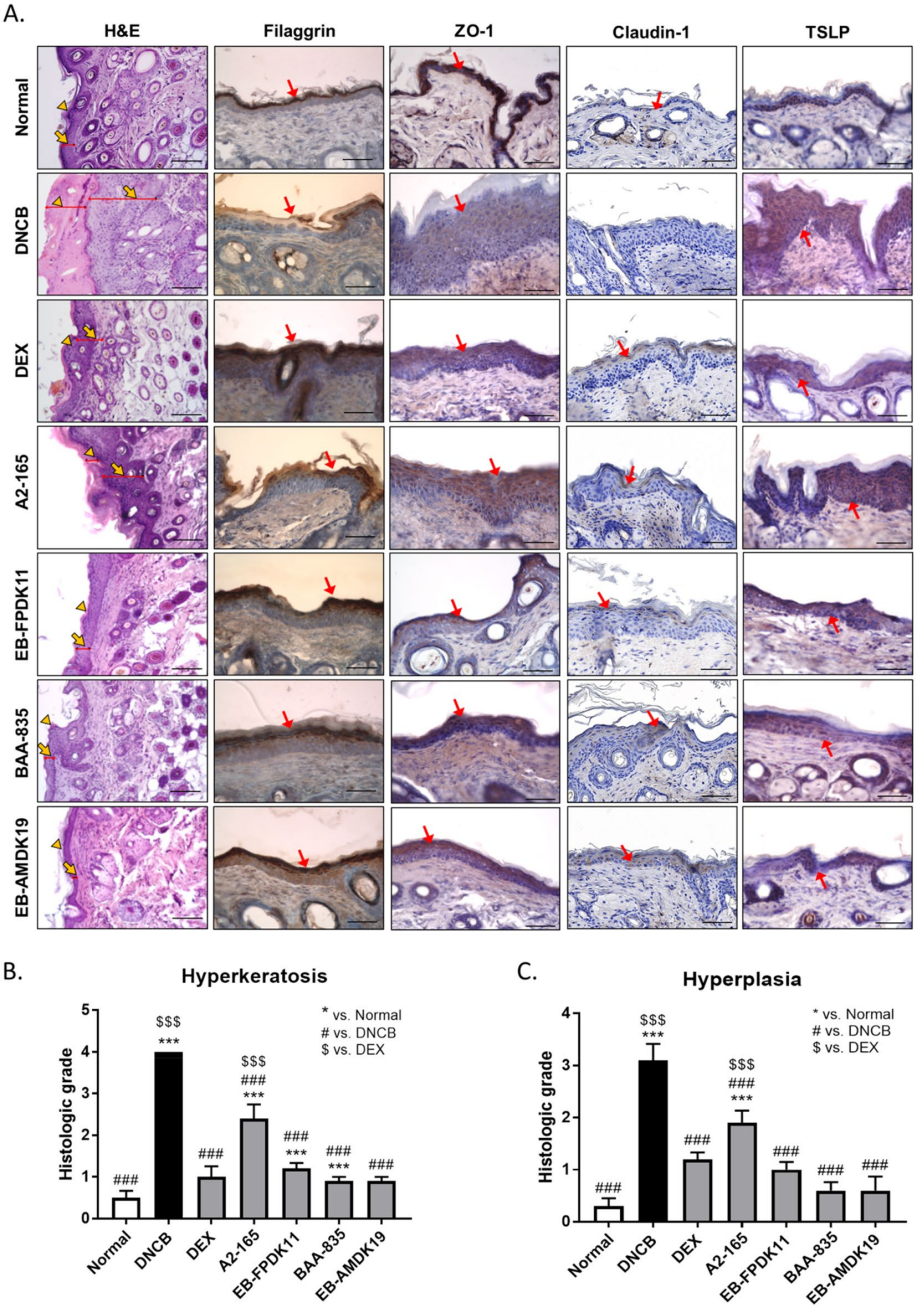
EB-FPDK11 and EB-AMDK19 enhance skin barrier proteins in AD-like lesion in NC/Nga mice. (**A**) The first lane: H&E staining of dorsal skin lesions. The yellow arrowheads indicate hyperplasia, and the yellow arrows indicate hyperkeratosis. The 2nd–4th lane: Immunohistochemical staining of filaggrin, ZO-1, claudin-1, and TSLP, respectively in dorsal skin lesions in DNCB-induced NC/Nga mice. Dark brown regions (the red arrows) indicate positively stained cells. Scale bar, 50 µm. (**B**, **C**) The graphs represented as (**B**) Hyperkeratosis (**C**) Hyperplasia, histological grades were scored as follows: 0, normal thickness; 1, two times normal thickness; 2, three times normal thickness; 3, four times normal thickness; or 4, greater than four times normal thickness. Data are presented as mean ± SEM of changes in values. *P < 0.05; **P < 0.01; ***P < 0.001; *versus normal group; ^#^versus DNCB group; ^$^versus DEX group.

In this regard, we performed histologic staining to determine the effects of the administration of bacterial strains on atopic skin lesions. H&E staining revealed hyperkeratosis, hyperplasia, and AD lesions following DNCB application (Fig. 4). The DNCB group showed a 3.6-fold increase in epidermal thickness compared with the normal group (Fig. 4A). The administration of the *A. muciniphila* and *F. prausnitzii* strains significantly reduced both hyperkeratosis (*P* < 0.001) and hyperplasia (*P* < 0.001) compared with the DNCB group (Fig. 4B, C). Next, we determined filaggrin expression using immunohistochemistry. Filaggrin expression in the skin was decreased in the DNCB group compared with the normal group, and was alleviated in the groups treated with the *A. muciniphila* or *F. prausnitzii* strains (Fig. 4A, Fig. S5A). We examined ZO-1 and claudin-1 expression levels using immunohistochemistry to assess TJ barrier function in the epidermis, because of the low expression of ZO-1 and claudin1 in skin lesion of AD patients^44,49^. The DNCB group showed a lower level of ZO-1 and claudin-1 proteins than the normal group, and the EB-FPDK11, BAA-835, and EB-AMDK19 treatment groups showed similar recovery to that of the normal group (Fig. 4A, Fig. S5B). TSLP activates dendritic cells to differentiate helper T cells into Th2 cells, which is critical to induce the symptoms of atopic dermatitis. When we determined the levels of TSLP in the skin, the levels were remarkably lower in the DEX, EB-FPDK11, BAA-835, and EB-AMDK19 groups than that in the DNCB group (Fig. 4A). Moreover, we determined the level of IL-4 cytokine in the skin using immunohistochemistry assay. It showed that the levels of IL-4 were increased in the DNCB induced group, however, they were significantly decreased in all groups administration with the EB strains, including dexamethasone, a positive control (Fig. S5C, D). Thus, these findings indicate that EB-FPDK11 and EB-AMDK19 administration was efficacious in reducing skin barrier abnormalities induced by DNCB by regulating filaggrin, ZO-1, Claudin-1, TSLP, and IL-4.

### Regulation of the balance between Th1 and Th2 cytokine by treatment with *F. prausnitzii *and *A. muciniphila* strains in DNCB-induced AD-like mice

AD is characterized by a predominant Th2 immune response that is derived from multiple cytokines, including IL-4 and IL-13^50^. In particular, IL-4 is a key Th2 cytokine for Th2 cell differentiation and IgE production^51^. IL-6 is secreted by activated T cells and macrophages and initiates the polarization of naïve CD4 + T cells to Th2 cells by inducing IL-4^52^. Recently, inhibition of IL-6 receptors was shown to improve AD in the clinical study^53^.

**Figure 5:**
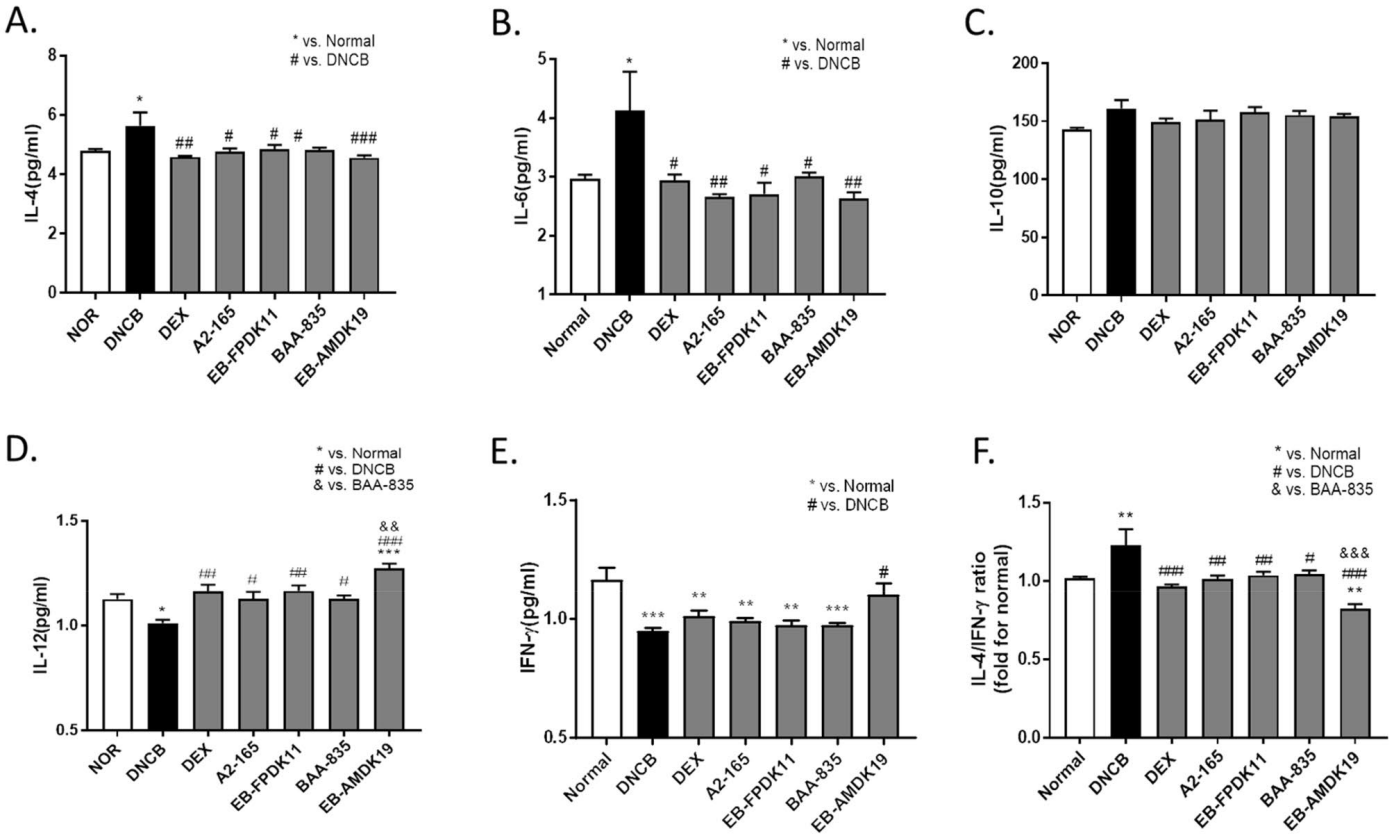
Oral administration of EB-FPDK11 and EB-AMDK19 attenuates Th2/Th1 cytokine imbalance in DNCB-induced AD like Nc/Nga mice. The Th2, Th1 related cytokines in serum (**A**) IL-4, (**B**) IL-6, (**C**) IL-10, (**D**) IL-12, (**E**) IFN-γ, and (**F**) IL-4/IFN-γ detected by mouse cytokine ELISA kit. Data are presented as mean ± SEM of changes in values. *P < 0.033; **P < 0.002; ***P < 0.001, *versus normal group; ^#^versus DNCB group; ^&^Versus BAA-835 group.

When we determined the changes in IL-4 and IL-6 cytokine levels related to Th2 responses, the respective cytokines in the serum were significantly elevated in the DNCB group compared with the normal group (both *P* < 0.05) but were significantly suppressed in the bacteria-administered groups (Fig. 5A, B). In contrast, there were hardly any changes in the levels of IL-10, which plays a suppressive role in AD^54^, in the treatment groups compared with the DNCB group (Fig. 5C). We also determined the respective levels of IL-12 and IFN-γ in relation to Th1 responses and found that they were significantly downregulated in the DNCB group compared with the normal group (*P* < 0.05 and *P* < 0.001, respectively) (Fig. 5D, E). The levels of IL-12 were significantly increased in all the treatment group compared with the DNCB group. In particular, the IL-12 level of the EB-AMDK19 group increased significantly higher than that of the BAA-835 group (*P* < 0.01) (Fig. 5D). Meanwhile, a significant increase in the level of IFN-γ was observed in the EB-AMDK19 group only (*P* < 0.05) compared with the DNCB group (Fig. 5E).

Recent studies on atopy and asthma indicate that total IgE levels are positively correlated with the ratio of IL-4/IFN-γ producing cells^55,56^. Additionally, Th2-cell-mediated immune responses are more prevalent than Th1-mediated immune responses and play an essential role in AD pathogenesis^57^. These findings suggest that the Th2/Th1 balance is related to atopic status^58–60^. Consequently, we investigated the effect of treatment with the bacterial strains on Th2/Th1 immune balance and analyzed the ratio of Th2 and Th1 related cytokines. The Th2/Th1 ratio, IL-4/IFN-γ, was increased by approximately 1.3-fold on average in the DNCB group compared with the Normal group (Fig. 5F). All the treated groups suppressed the increases in the Th2/Th1 ratiosThus, these results indicate that the administration of the *F. prausnitzii* and *A. muciniphila* strains may counteract the imbalance in the immune response induced by DNCB.

### Alleviation of DNCB-induced colon injury in NC/Nga mice treated with EB-FPDK11 and EB-AMDK19

**Figure 6:**
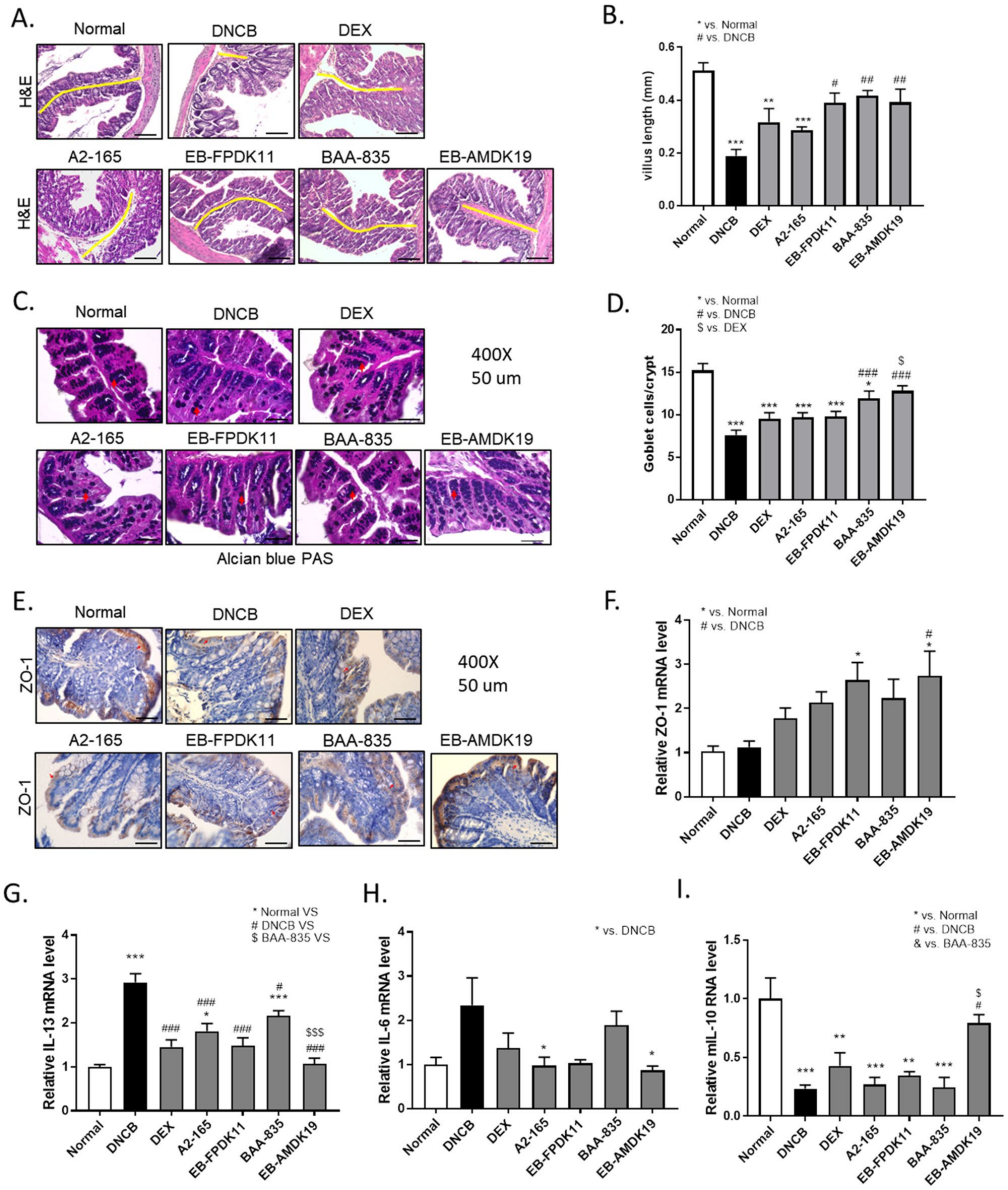
EB-FPDK11 and EB-AMDK19 facilitate intestinal epithelial barrier function maintenance from DNCB-induced colon injury. (**A**) H&E staining of the large intestinal tissues from DNCB-induced AD-like Nc/Nga mice. The yellow lines indicate the villi length. Scale bar, 100 µm. (**B**) Large intestine villi length was determined by measuring vertically well-oriented crypt villi from H&E stained sections. (**C**) Alcian blue PAS staining of the large intestinal tissues, the red arrows indicate positively stained goblet cells. (**D**) Quantitative analysis of the number of goblet cells in the crypt of the colon. (**E**) The expression of ZO-1 proteins in the colon tissue were analyzed using immunohistochemistry staining. Scale bar, 50 µm. (**F**) Relative expression of ZO-1 mRNA expression in the colon tissue was determined by quantitative PCR. (**G**–**I**) Transcript levels of Th2 related cytokine genes, mIL-13 (**G**), mIL-6 (**H**), and mIL-10 (**I**) were determined by quantitative PCR. Data are presented as mean ± SEM of changes in values. *P < 0.05, **P < 0.01, ***P < 0.001; *versus normal group; ^#^versus DNCB group; ^$^versus DEX group; ^&^versus BAA-835 group.

We examined the colonic morphology and gut barrier function to investigate the effects of the bacterial strains on DNCB-induced gut barrier dysfunction (Fig. 6A–F). H&E staining of the colon showed that the DNCB treatment induced severe mucosal damage: the villous shape was irregular, and the length was shortened significantly due to inflammation compared with the normal group (DNCB: 0.19 ± 0.02 mm versus normal: 0.51 ± 0.03 mm, *P* < 0.001) (Fig. 6B). The villus length appeared to be recovered in the treatment groups compared with the DNCB group and was significant only in the EB-FPDK11 (*P* < 0.05), BAA-835 (*P* < 0.01), and EB-AMDK19 (*P* < 0.01) groups (Fig. 6A, B). Goblet cells are columnar epithelial cells responsible for the secretion of mucins and function as the gut barrier, which protects the epithelium^61^. Thus, we investigated how the goblet cells were affected in the study groups. After staining the colonic tissues with Alcian blue/PAS, we found that the number of goblet cells in the DNCB group was reduced twofold (*P* < 0.001) compared with that of the normal group, while the numbers were significantly increased by 1.6-fold in the BAA-835 group (*P* < 0.001) and by 1.7-fold I the EB-AMDK19 group (*P* < 0.001) compared with that of DNCB group (Fig. 6C, D). Next, we examined the expression level of ZO-1 protein using immunohistochemistry in the colon and found that the level was apparently increased in the EB-AMDK19 group only (Fig. 6E). To verify this, we also measured the expression of ZO-1 at the transcriptional level and found that it tended to increase in the DEX, A2-165, and BAA-835 groups or was significantly increased in the EB-FPDK11 and EB-AMDK19 groups and was, thus, in good agreement with the results in Fig. 5E (Fig. 6F). These results suggested that the administration of EB-FPDK11 or EB-AMDK19 not only attenuated the DNCB-induced colon injury but also improved the intestinal barrier dysfunction caused by allergenic compounds. (Fig. 6A–F). Lastly, we investigated the effects on the gut mucosal immune cells by quantifying the levels of anti-inflammatory (IL-10) or pro-inflammatory (IL-6 and IL-13) cytokines in the colon by qPCR. The relative mRNA level of IL-13 in the DNCB group was increased by 2.9-fold compared with the normal group and was statistically significant (*P* < 0.001), and significantly lowered in the treatment groups compared with the DNCB group (*P* < 0.001 for the BAA-835 group and *P* < 0.001 for the other treatment groups), with the lowest level in the EB-AMDK19 group (Fig. 6G). The IL-6 level also showed a similar trend to IL-13 but this was only significant in the A2-165 and EB-AMDK19 groups (both *P* < 0.01) (Fig. 6H). In contrast, the relative mRNA levels of IL-10 were significantly decreased by 77% ± 3% in the DNCB group compared with the normal group (*P* < 0.001) and was upregulated in the EB-AMDK19 group only, showing a 3.39-fold increase (*P* < 0.05) (Fig. 6I).

Based on the results obtained in this study, we concluded that the administration of *F. prausnitzii* and *A. muciniphila* strains may play a therapeutic role in AD-like symptoms, among which EB-AMDK19 appeared to have the highest potential, by controlling intestinal barrier functions and immune responses in the gastrointestinal tract.

## Discussion

Atopic dermatitis (AD) is a chronic inflammatory skin disease with a high recurrence rate. Corticosteroids (e.g. Dexamethasone) are widely used for the treatment of AD, but their effects are limited and adverse effects from long-term use have a great impact on people’s lifestyles^62^. Because of the limited effectiveness of these treatments, new strategies for recovery from AD lesions are continually being explored.

Gut microbiota and their repertoire of biochemical reactions contribute to many aspects of host health, including metabolism, immunity, development, and behavior. Microbial dysbiosis, which is an imbalance of the microbial community, can contribute to the development of numerous diseases^63^. From studies on the health beneficial effects of probiotics, most of the materials used are live bacteria, and of these, *Lactobacillus* and *Bifidobacterium* called traditional probiotics, have been the most frequently investigated^64^, while there have been limited studies of next-generation probiotics such as *A. muciniphila* and *F. prausnitzii* in AD. However, next-generation probiotics have emerged as new preventive and therapeutic tools, since traditional probiotics generally show marginal effects, and the results were not consistent. In recent meta-analytic studies, it has been evident that harmonized intestinal microbes can modulate the development of allergic diseases including AD. For example, a significant depletion in members of the *Clostridium* cluster IV, *F. prausnitzii*, and *A. muciniphila* and an increase of the relative abundance of *Enterobacteriaceae* in the gut microbiota of the children with AD compared to the healthy controls has been reported^26^. In addition, an analysis of gut microbiota in patients with immune diseases, such as asthma, suggested that *F. prausnitzii* and *A. muciniphila* have a negative correlation with symptoms^25^. However, until now, the mechanisms by which these bacteria may have therapeutic effects remain unclear and their clinical efficacy has not yet been clearly demonstrated.

In present study, we investigated the effects of *F. prausnitzii* and *A. muciniphila* on AD symptoms by using a DNCB-induced AD-like NC/Nga mouse model. The previous researches clearly showed that skin exposure to DNCB leads to the dysbiosis of the gut microbiome related to AD development^65,66^. Also, they showed that the DNCB-induced AD was ameliorated by restoring the gut microbiome profile. A clinical study observed statistical significance of a higher incidence of skin disease in patients with inflammatory bowel disease (IBD), which suggested that intestinal dysbiosis was associated with an increased risk of inflammatory skin disease^15^ In addition, intestinal dysbiosis causes the loss of gut barrier function, leading to a state permeable to toxins, SCFAs, and bacterial metabolites, reaching the circulatory system. The consequent outcomes accumulate in the skin and finally impair skin barrier function. It suggests that the specific gut microbiome (such as *A. muciniphila* and *F. prausnitzii*) with metabolites (such as functional genes and SCFAs), which have the potential to improve the gut barrier function, can be proposed as one of the effective therapeutic options for AD^17,67^.

Our study demonstrated that the administration of specific *A. muciniphila* and *F. prausnizii* strains could significantly alleviate the symptoms of AD via modulation of the immune response and gut barrier function. *A. muciniphila* and *F. prausnitzii* are thought to exert its anti-inflammatory effects through its metabolites, which control the genes that regulate gut function, especially in host intestinal epithelial cells. *A. muciniphila* and *F. prausnitzi*i has been found to increase anti-inflammatory immune responses by secreting short chain fatty acids (SCFAs) such as propionate, acetate, and butyrate^68^. The SCFAs can directly promote T-cell differentiation into T cells producing IL-17, IFN-γ, and IL-10^69^ and suppress the cytokine-stimulated production of pro-inflammatory mediators including TNF-α, IL-6, and NO^71^, and cause an impaired G-protein-coupled receptor (GPR) 41 mediated Th2 polarization in allergic airway inflammation^71^. In particular, butyrate represents a significant energy source for the host colonocytes and also consume oxygen, contributing to anaerobic conditions and gut barrier integrity^68^. Propionate is well known to improve intestinal barrier function and reduce inflammation and oxidative stress via the STAT3 signaling pathway^72^. We observed that *F. prausnitzii* produced butyrate as a major metabolite at the expense of acetate and *A. muciniphila* produced acetate and propionate (Table S3). Some literature described that A. muciniphila could produce succinate^73^, and the propionate was made via the succinate-metabolic pathway^74^. Interestingly, the consumption of succinate by EB-AMDK19 was higher than ATCC BAA-835, although both BAA-835 and EB-AMDK19 showed similar levels of succinate production in the absence of cobalamin (Table S3). In our results, the strain-specific properties indicate that they may have different anti-inflammatory effects since succinate can stabilize hypoxia-inducible factor 1α, which, in turn, promotes transcription of IL-1β^75^. To confirm this, it needs to define how the SCFAs change by administering strains of *A. muciniphila* or *F. prausnitzii* in AD-induced animal models.

In this study, we also found that DNCB treatments on dorsal skin induced local allergic inflammation, systemic sensitization, and gut barrier impairment with imbalance of immune homeostasis such as Th1/Th2 balance. Oral administration of EB-AMDK19 and EB-FPDK11 modulated the immune response, leading to suppression of Th2-related cytokines and increase of Th1-related cytokines (Fig. 5). These results suggest the potential EB-FPDK11 and EB-AMDK19 treatment to regulate the immune-modulatory response by restoring the Th2/Th1 dysbalance and gut barrier impairment. The improvement in overall AD-like symptoms by administering the EB-FPDK11 strain was superior to that of its type strain, A2-165. Meanwhile, the EB-AMDK19 strain seemed to have more substantial impacts on the improvement of AD than the type strain, BAA-835, thereby restoring the immune responses and gut barrier functions. These results may support that the efficacy of probiotic strains is both strain-specific and disease-specific.

On the other hand, we performed an experiment using germ-free mice to determine whether the administered EB-AMDK19 reached the gut alive, where it induced immune-modulatory responses. We analyzed the *A. muciniphila* abundance in the cecum, the colon, and the feces after oral administration within 72 h at an amount of 1X10^8^ CFUs/head. Its abundance in each organ was found to sharply increase to about 1 × 10^10^ CFUs/head (Fig. S6A). In addition, the abundance in the feces was maintained from the 3rd day until the end of the study (17th day). These results indicate that EB-AMDK19 reached and stably proliferated in the gut (Fig. S6B). We also investigated if EB-AMDK19 alone can exert the immunomodulatory effects and thus analyzed the myeloid subpopulation and T cell polarization in the mLN and the spleen by administering the strain into normal GF mice. Fig. S7 showed that no apparent change in immune response was seen with EB-AMDK19 administration. These results suggest that administration of *F. prausnitzii* or *A. muciniphila* is less likely to induce excessive immune response and is safe in healthy conditions without DNCB treatment.

In summary, our findings show, for the first time, that the oral administration of *F. prausnitzii* and *A. muciniphila* significantly improved the AD-like symptoms. In particular, the improvements with EB-AMDK19 or EB-FPDK11 appeared to be superior to BAA-835 or A2-165, respectively. Mainly, EB-FPDK11 and EB-AMDK19 seemed to have similar or better efficacies in terms of their anti-atopic effect compared with that of dexamethasone, a widely used steroid drug for AD. Therefore, the supplementation with *F. prausnitzii,* EB-FPDK11 or *A. muciniphila,* EB-AMDK19 could be a novel therapeutic option for patients with AD. However, clinical researches should be necessary to confirm the efficacies of EB-FPDK11 and EB-AMDK19 in humans.

## Supplementary Information

Supplementary Information.

## Data Availability

The datasets used and/or analysed during the current study available from the corresponding author on reasonable request.
