# Supplementary material for: Oral administration of Faecalibacterium prausnitzii and Akkermansia muciniphila strains from humans improves atopic dermatitis symptoms in DNCB induced NC/Nga mice
Source: Sci Rep. 2022 May 5;12:7324. doi: 10.1038/s41598-022-11048-4 (PMC9072367; doi:10.1038/s41598-022-11048-4)
Supplement: Supplementary file 1 — Supplementary Information. [file 41598_2022_11048_MOESM1_ESM.docx]

**Oral administration of *Faecalibacterium prausnitzii* and *Akkermansia muciniphila* strains from humans improves atopic dermatitis symptoms in DNCB induced NC/Nga mice**

Yoonmi Lee^1^, Hye Rim Byeon^1^, Seo Yul Jang^1^, Moon-Gi Hong^1^, Dohak Kim^1^, Dokyung Lee^1^, Joo-Hyun Shin^1^, Yesol Kim^2^, Seung Goo Kang^2^, and JaeGu Seo^1,*^

^1^R&D Center, Enterobiome Inc., 814 Siksa-dong, Ilsandong-gu, Goyang-si 10326, Korea;

^2^Institute of Bioscience & Biotechnology, Kangwon National University, Chuncheon

*Correspondence : JaeGu Seo^1^ (email:jgseo@enterobiome.com)


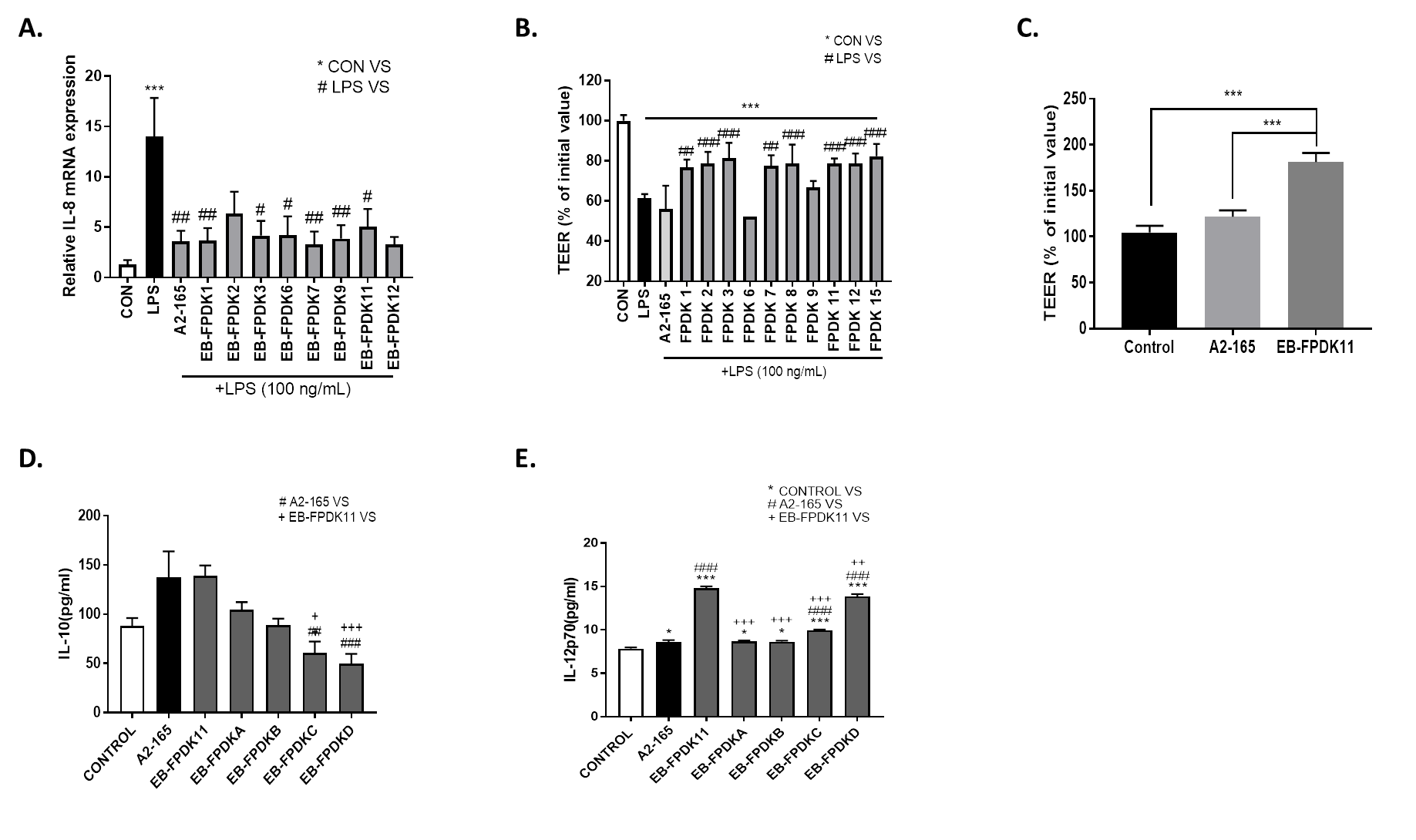


**Supplementary Fig S1**. *F. prausnitzii* EB-FPDK11 selected from *F. prausnitzii* strain library. (A) LPS-induced pro-inflammatory cytokine, mIL-8 were measured using quantitative real-time PCR in HT-29 cell line (B, C) TEER changes in Caco-2 cell treated with LPS (B), or without LPS (C), for 24 h., (D, E) Cytokine secretion by mouse BMDC. IL-10 (D) and IL-12p70 were measured in the supernatant of BMDCs after 24 h of incubation with *F. prausnitzii* strains by mouse cytokine ELISA kit ; All experiments were treated *F. prausnitzii* at 1X10^7^ CFUs/well in 24w plate. Data are presented as mean±SEM of changes in values. * :*P <* 0.05, **:*P <* 0.01, ***:*P <* 0.001


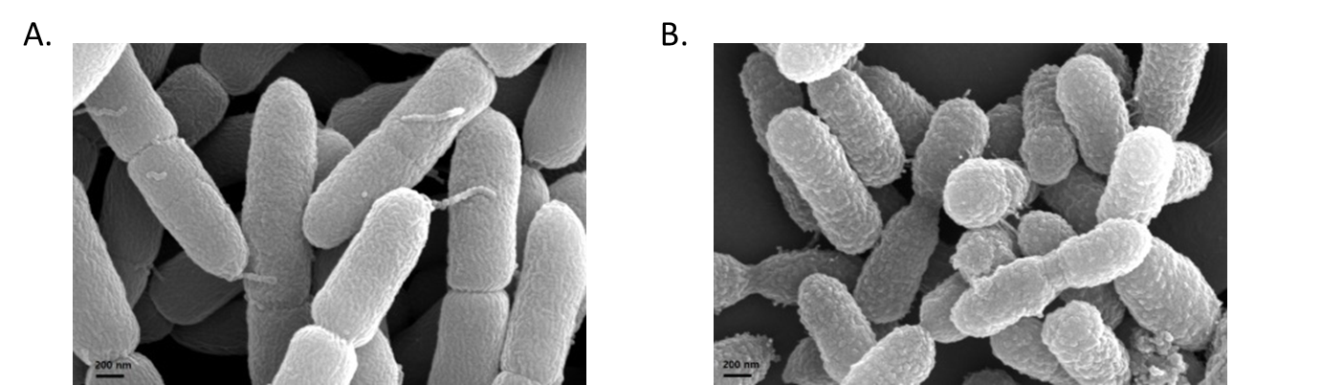


**Supplementary Fig S2**. (A) SEM image of *F. prausnitzii,* EB-FPDK11, 3000KX (B) SEM image of *A. muciniphila,* EB-AMDK19, 3000KX.


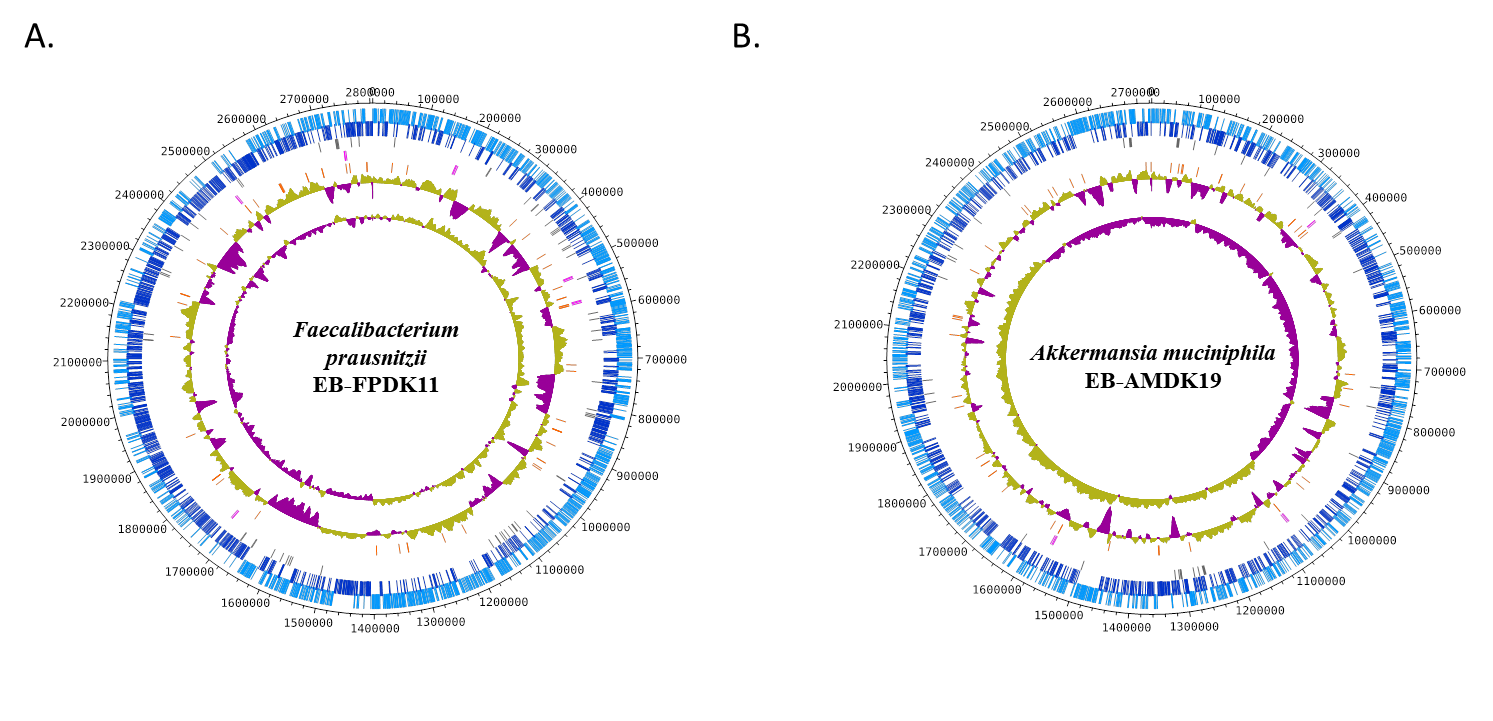


**Supplementary Fig S3**. Circular map of *F prausnitzii* EB-FPDK11 (2.8 Mb chromosome) and *A. muciniphila* EB-AMDK19 (2.7 Mb chromosome). Seven tracks were plotted on the map: Track 1 (light blue; outset), forward-strand coding CDS; Track 2 (blue), reverse-strand coding CDS; Track 3 (gray): pseudogenes; Track 4 (light purple), rRNAs; Track 5 (orange), tRNAs; Track 6 (light green and purple), G + C content; and Track 7 (light green and purple): GC skew. DNAPlotter^1^ as used to draw chromosome topology of genome.


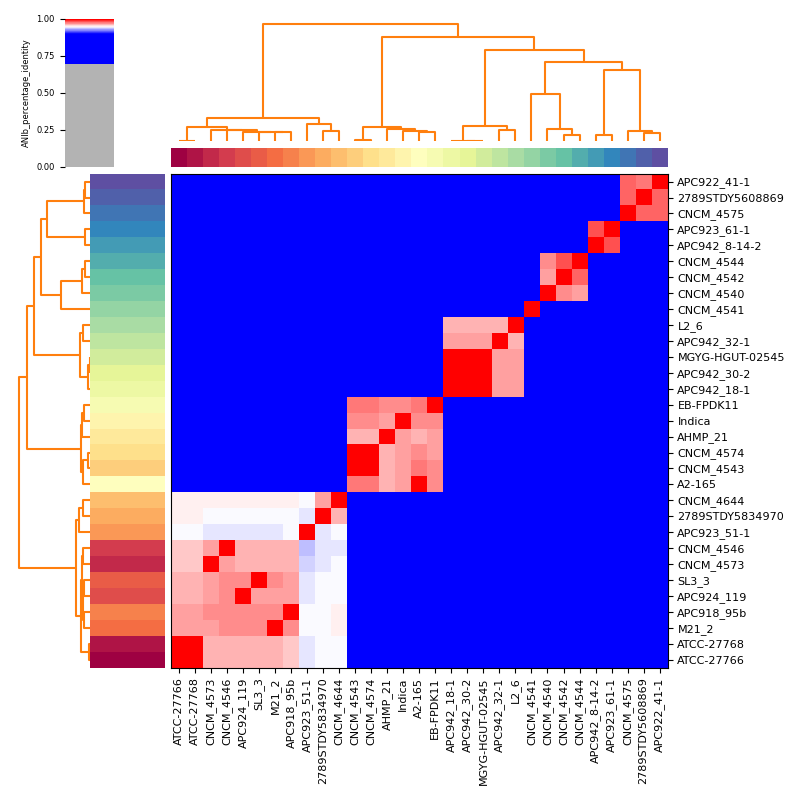


**Supplementary Fig S4.** Genome-to-genome identity of *Faecalibacterium prausnitzii* genomes. Color bars representing correlation coefficients (0–1.0) are shown with the color scale based on respective percentage identity. Average nucleotide identity (ANI) values were calculated to evaluate evolutionary distance using pyani version 0.2.7; option “-m ANIb”^2^. Complete or draft genome sequences were downloaded from the NCBI genome database [http://www.ncbi.nlm. nih.gov/genome/; accession nos. GCF_003324115 (ATCC-27766); GCF_003324185 (ATCC-27768); GCF_000154385 (M21_2); GCF_003312465 (APC918_95b); GCF_003287475 (APC924_119); GCF_000209855 (SL3_3); GCF_002549945 (CNCM_4573); GCF_002549935 (CNCM_4546); GCF_003287405 (APC923_51-1); GCF_001406255 (2789STDY5834970); GCF_002550015 (CNCM_4644); GCF_002734145 (A2-165); GCF_002549855 (CNCM_4543); GCF_002549985 (CNCM_4574); GCF_002550035 (AHMP_21); GCF_002586945 (Indica); GCF_003287505 (APC942_18-1); GCF_003293635 (APC942_30-2); GCF_902388275 (MGYG-HGUT-02545); GCF_003287485 (APC942_32-1); GCF_000210735 (L2_6); GCF_002549775 (CNCM_4541); GCF_002549755 (CNCM_4540); GCF_002549895 (CNCM_4542); GCF_002549905 (CNCM_4544); GCF_003287415 (APC942_8-14-2); GCF_003287495 (APC923_61-1); GCF_002549975 (CNCM_4575); GCF_001406355 (2789STDY5608869); GCF_003287455 (APC922_41-1)].


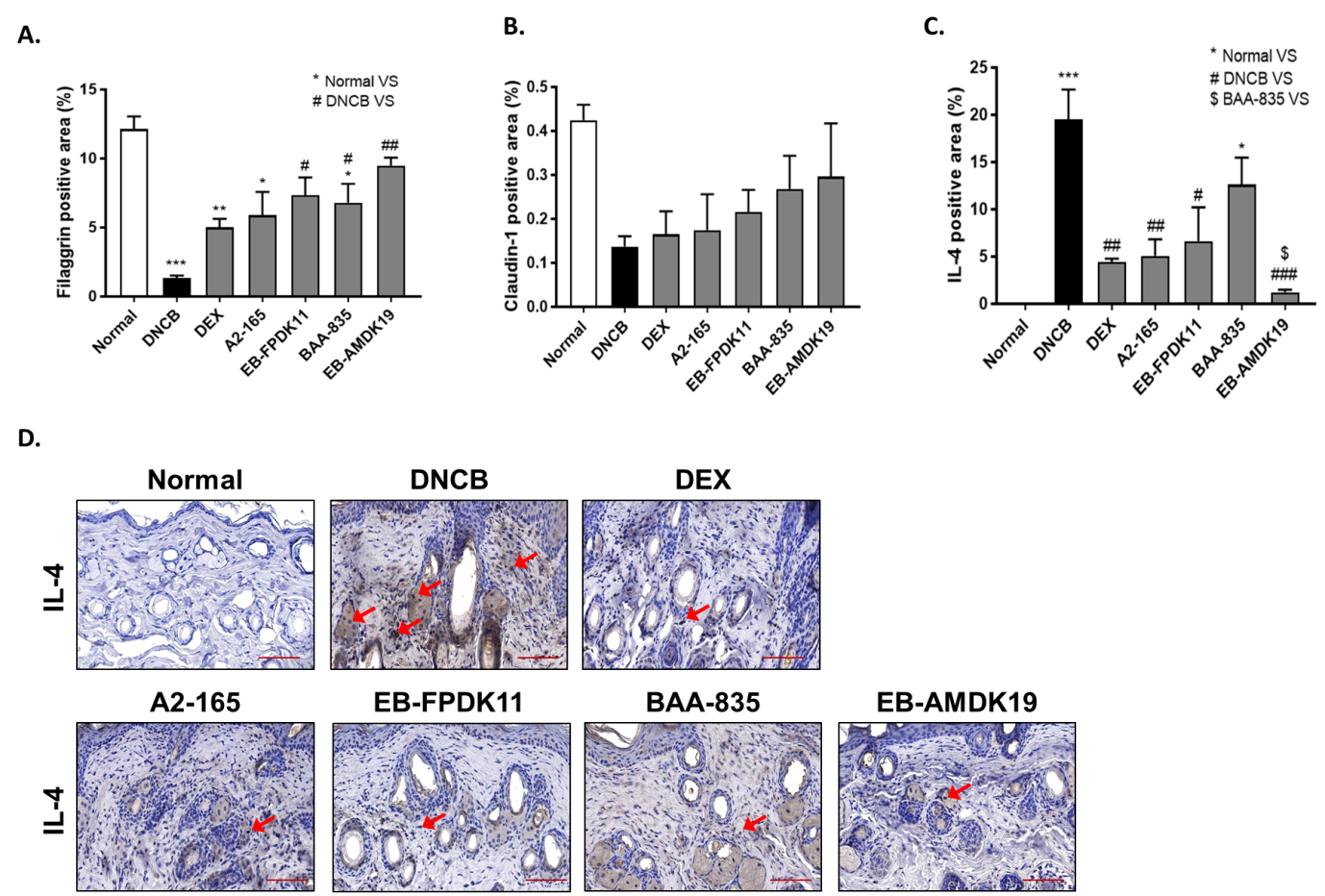


**Supplementary Fig S5.** EB-FPDK11 and EB-AMDK19 enhance skin barrier proteins and suppresses the IL-4 cytokine levels in AD-like lesion in NC/Nga mice**.** (A) Filaggrin, skin barrier proteins, (B) Claudin-1, skin barrier proteins, and (C) IL-4, Th2 related cytokine; Quantification of positive staining area was analyzed by densitometry with 3 samples from each group. (D) Immunohistochemical staining of IL-4 of dorsal skin lesions. Dark brown regions (the red arrows) indicate positively stained cells. Scale bar, 50 mm (B, C) Data are presented as mean±SEM of changes in values. * :*P <* 0.05, **: *P <* 0.01, ***: *P <* 0.001, * vs. normal group; # *vs*. DNCB group; $ *vs*. DEX group.


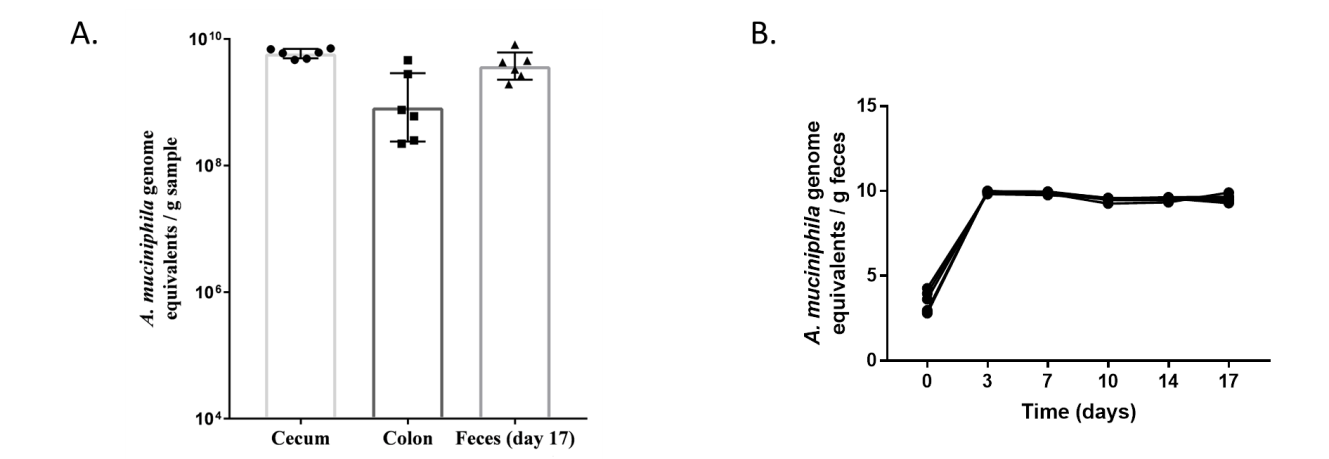


**Supplementary Fig S6.** *A. muciniphila* reach and stably proliferate in the gut. (A) *A. muciniphila* levels in the cecum, the colon, and the feces from germ-free mice (B) *A. muciniphila* levels in the feces at several time point. Germ-free mice were inoculation with the EB-AMDK19 at an amount of 1X10^9^ CFUs/head within 72 h and kept in a germ-free isolator. Fecal samples were collected at different time points for analysis of *A. muciniphila* abundance. At the end day (17^th^ day), the cecum, the colon, and the feces were obtained and frozen at -80. For analysis of *A. muciniphila* abundance in the cecum, the colon, and the feces, bacterial DNA was extracted by QIAamp PowerFecal Pro DNA Kit (12830-50, Quiagen, Germany). After adjusting the DNA concentration, levels of *A. muciniphila* was assessed by qPCR with *A. muciniphila* species primer (Forward 5′-CAGCACGTGAAGGTGGGGAC-3′; Reverse 5′-CCTTGCGGTTGGCTTCAGAT-3′). PCR was run with SYBR® Green real-time PCR Master Mix on a QuantStudio 3 real-time PCR system (Applied Biosystems, USA). The abundance of *A. muciniphila* was calculated as copies per gram feces. Statistical analyses and plots were generated with GraphPad Prism version 7.04.


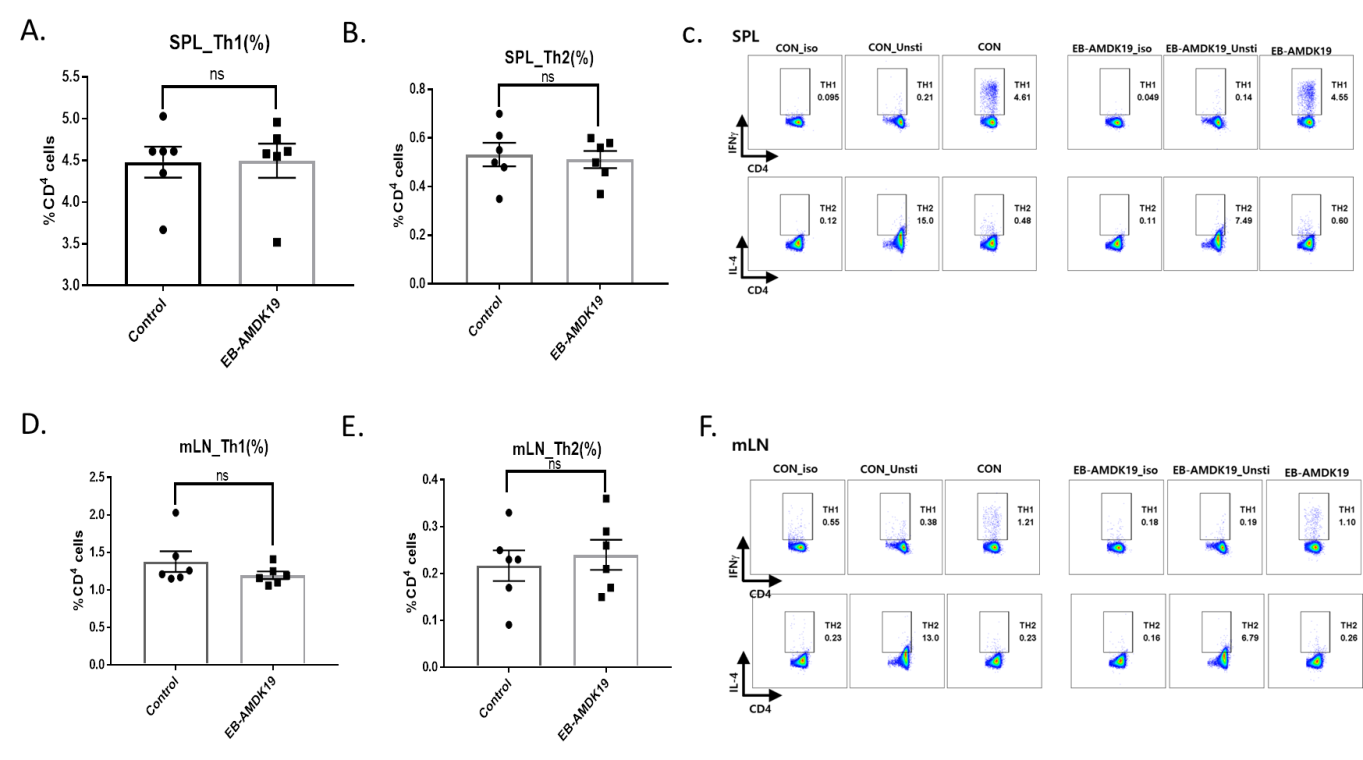


**Supplementary Fig S7**. Administration of *A. muciniphila* does not change immunogical profile in healthy condition. Th1 and Th2 polarization in the spleen (A-C) and mLN (D-F) were analyzed after EB-AMDK19 was administered to germ-free mice. Six-week-old mice were treated with the EB-AMDK19 in a germ-free isolator as described in Supplementary Fig S7. On day 17 mice were sacrificed, the spleen or mLN was removed and the single cell suspension was prepared for further FACS staining. Each subset of lymphoid T cells was analyzed by flow cytometry using a FACSAria flow cytometer (Becton Dickinson) after staining with mAbs specific for CD3, CD4, IL-4, or IFNγ (eBiosciences and BD Biosciences). Data were analysed with FlowJo software (Tree Star, Inc). ns:no significant.

**Supplementary Table S1. Comparison of whole genome sequences between type strain and isolates**


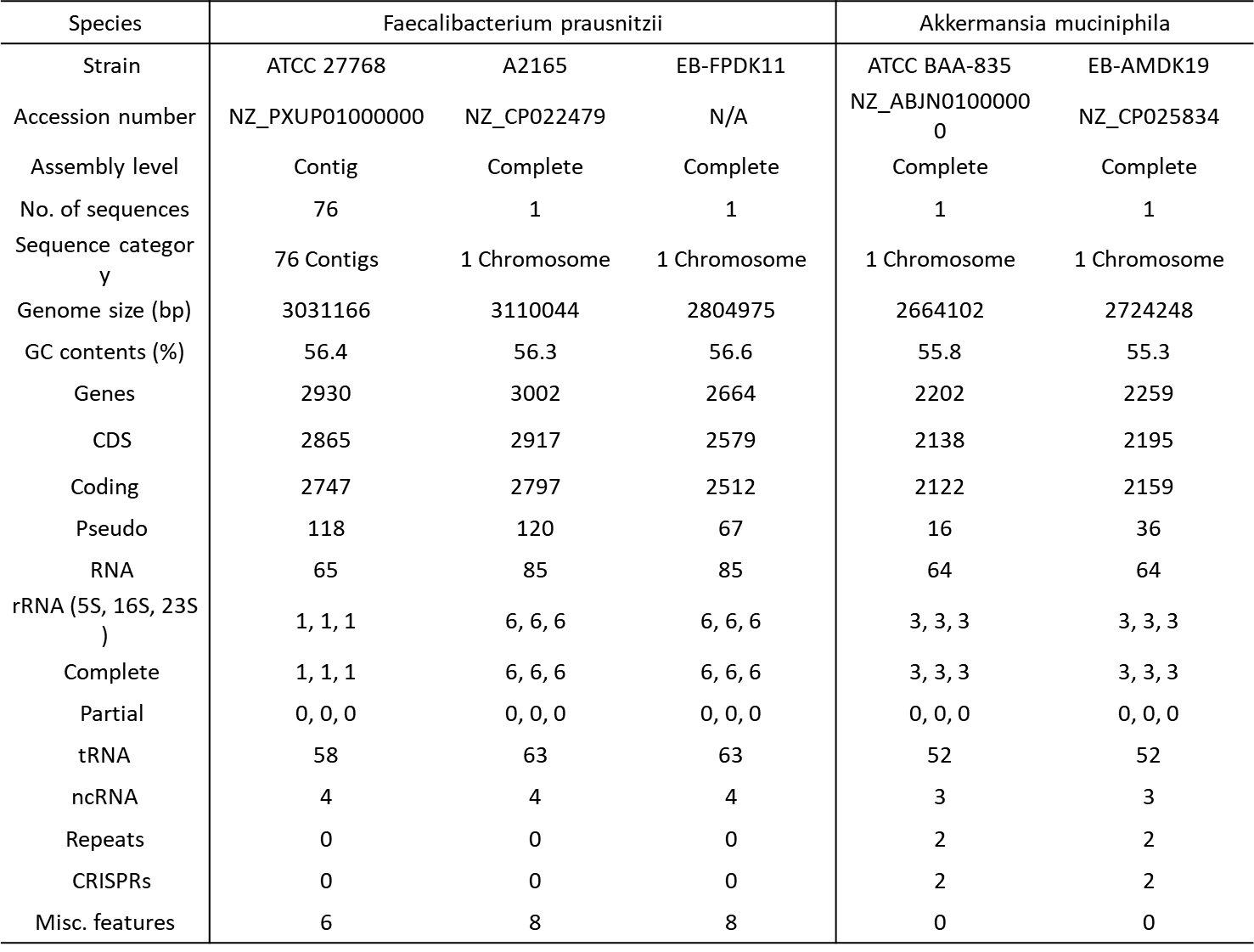


| Flow | H_2_: 40 mL/min, Air: 350 mL/min |
| --- | --- |
| Injector temp. | 240℃ |
| Detector temp. | 250℃ |
| Oven temp. | 40℃ (hold 2 min) → 65℃/10 min (hold 2 min) → 240℃/10 min (hold 5 min) |
| Injection vol. | 2 μL |
| Split ratio | 20:1 |

**Supplementary Table S2. The conditions of chromatographic analysis**

**Supplementary Table S3. Short Chain Fatty Acid formed and/or utilized by F. prausnitzii strains and A. muciniphila strains**

Reference

1 Carver, T., Thomson, N., Bleasby, A., Berriman, M. & Parkhill, J. DNAPlotter: circular and linear interactive genome visualization. *Bioinformatics* **25**, 119-120, doi:10.1093/bioinformatics/btn578 (2009).

2 Pritchard L, G. R., Humphris S, Elphinstone JG, Toth IK. Genomics and taxonomy in diagnostics for food security: soft-rotting enterobacterial plant pathogens. *Analytical Methods* **8**, doi:DOI: 10.1039/C5AY02550H (2016).
